# A Common Kinetic Property of Mutations Linked to Episodic Ataxia Type 1 Studied in the Shaker Kv Channel

**DOI:** 10.3390/ijms21207602

**Published:** 2020-10-14

**Authors:** Juan Zhao, Dimitri Petitjean, Georges A. Haddad, Zarah Batulan, Rikard Blunck

**Affiliations:** Department of Physics, Université de Montréal, Montréal, QC H3C 3J7, Canada; Juan.zhao.work@gmail.com (J.Z.); Dimitri.petitjean.stm@gmail.com (D.P.); Georges.anthony.haddad@gmail.com (G.A.H.); zbatulan@gmail.com (Z.B.)

**Keywords:** voltage-gated potassium channel, episodic ataxia, gating, electrophysiology

## Abstract

(1) Background: Episodic ataxia type 1 is caused by mutations in the *KCNA1* gene encoding for the voltage-gated potassium channel Kv1.1. There have been many mutations in Kv1.1 linked to episodic ataxia reported and typically investigated by themselves or in small groups. The aim of this article is to determine whether we can define a functional parameter common to all Kv1.1 mutants that have been linked to episodic ataxia. (2) Methods: We introduced the disease mutations linked to episodic ataxia in the drosophila analog of Kv1.1, the Shaker Kv channel, and expressed the channels in Xenopus oocytes. Using the cut-open oocyte technique, we characterized the gating and ionic currents. (3) Results: We found that the episodic ataxia mutations variably altered the different gating mechanisms described for Kv channels. The common characteristic was a conductance voltage relationship and inactivation shifted to less polarized potentials. (4) Conclusions: We suggest that a combination of a prolonged action potential and slowed and incomplete inactivation leads to development of ataxia when Kv channels cannot follow or adapt to high firing rates.

## 1. Introduction

Episodic ataxia type 1 (EA1) is an autosomal dominant K^+^ channelopathy. EA1 manifests itself as myokymia and episodes of cerebellar dysfunction with spastic contraction of skeletal muscles and loss of motor coordination, vertigo, migraine and ataxia. It is occasionally associated with epilepsy, dystonia, hemiplegic migraine, myasthenia and intermittent coma [1,2]. The episodes can last for seconds to minutes. EA1 is caused by mutations in the *KCNA1* gene encoding for Kv1.1.

**Figure 1:**
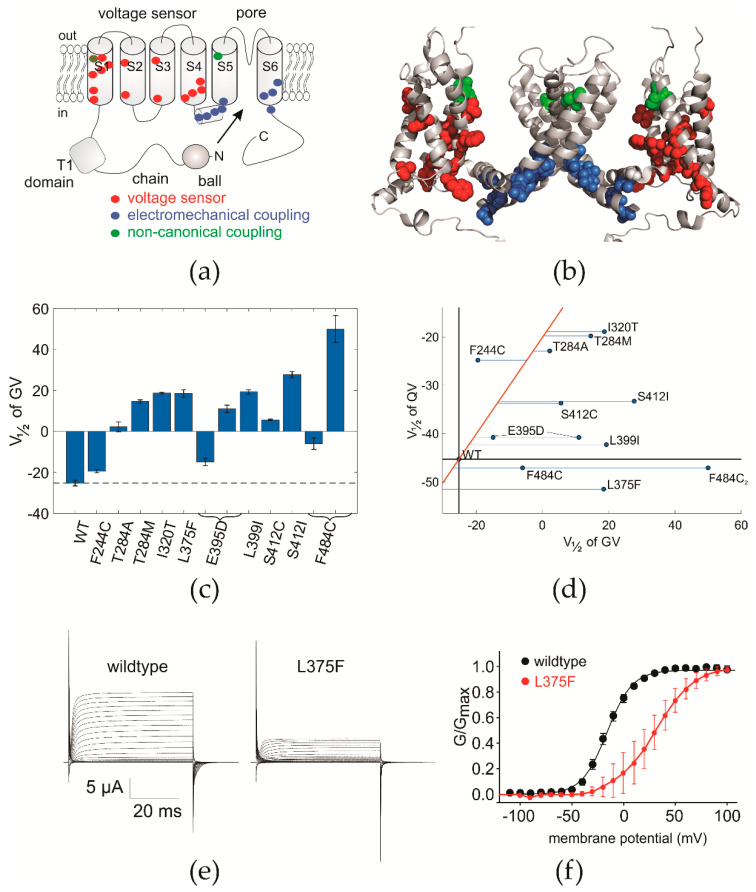
(**a**) Topology of Kv1.1 and location of the identified mutations causing Episodic ataxia type 1 (EA1). Red denotes the voltage sensor mutations, blue the mutations in the region of electromechanical coupling and green those in the position for non-canonical coupling. (**b**) Position of the mutations in the 3D structure (PDB: 3LUT). Colors coded as in (**a**). (**c**) Midpoint of activation for the tested mutants. The dashed line indicates wildtype. Midpoints and their standard error were obtained from fit to sigmoidal curves (see Materials and Methods). E395D and F484C had to be fitted to a convolution of 2 sigmoidal curves (*n* = 3–6). (**d**) Midpoints of activation for pore opening (GV) and voltage sensor movement (QV). The red line shows a parallel shift of both QV and GV. The arrows indicate a separation (*green*) or convergence (*red*) of QV and GV. (**e**) Current traces elicited from wildtype and Shaker-L375F upon response to depolarizing pulses from a holding potential of −90 mV to voltages between −120 and +100 mV. (**f**) Current voltage relationship of wildtype and the furthest shifted of Shaker-L375F.

In neurons, Kv1.1 (KCNA1) is expressed mainly in the axon and the distal axon initial segment but is also found in the cell body and dendrites [3,4]. It may form heteromers with Kv1.2 and Kv1.4. Kv1.1 channels are responsible for the repolarization of the membranes of excitable cells after depolarization through sodium and calcium influx. They also adjust the excitability of these membranes. As a member of the delayed outward rectifiers, the tetrameric Kv1.1 has the typical topology with six transmembrane helices S1–S6, where S1–S4 form four peripheral voltage sensors around a single central ion conducting pore domain formed by the four S5–S6 helices (Figure 1a,b). 

Thirty years of intense biophysical studies since the first cloning of the drosophila analog of Kv1 [5] resulted in a thorough understanding of the molecular mechanisms underlying Kv channel function. The mutations linked to EA1 have been the topic of several of these biophysical studies, characterizing the underlying molecular mechanism [6,7,8,9,10,11,12]. Still, how the effects on the molecular level lead to the development of EA1 remains unclear. On first sight, the EA1 mutations seem to be distributed arbitrarily throughout the transmembrane region of the Kv1.1 channel (Figure 1a), however, in the 3D structure (Figure 1b), it becomes apparent that they are cumulated in two regions of the channel, the voltage sensor domain and the region for electromechanical coupling. This suggests that all mutants may share one common mechanism or phenotype that is responsible for the development of episodic ataxia. If we can identify this common property, it will help us on the path to decipher how the Kv1.1 dysfunction leads to EA1. We thus set out to study the biophysical properties of a group of EA1-linked mutations and compared the functional parameters in order to identify the common parameters. 

## 2. Results

### 2.1. Conductance-Voltage Relations Are Shifted to More Polarized Potentials

We constructed a number of Kv1.1 mutants in the *drosophila* analog Kv1 Shaker channel (Table 1, numbering in this article according to Shaker). According to their position in the 3D structure, the mutants can be classified with few exceptions into two groups, those that are in the voltage sensor and those that are in the region of electromechanical coupling, i.e. the energetic coupling between the voltage sensor and the pore (Figure 1a,b). The exception is S342I/C [13], which is located in the path of non-canonical coupling [7,14] and which we will discuss specifically below. Among the mutations in the voltage sensor, none affected any of the major cationic gating charges in the S4. R377C (the sixth gating charge in S4) is known not to express [15]. Three other EA1 mutants were located between the gating charges (V369I, F373V, L375F) while the remaining mutations in the voltage-sensing domain were distributed over the surrounding S1–S3 (Figure 1a,b). 

The mutations in the region known to be responsible for the electromechanical coupling between voltage sensor and pore were located in the S4–S5 linker and the N-terminal S6. Annealing of the S4–S5 linker to the C-terminal S6 is essential to facilitate energy transfer from the voltage sensor to the pore region [35,36,37,38,39]. Since all these mutants cause episodic ataxia (type 1) and most are concentrated in two regions, we aimed to verify whether we find a common underlying mechanism on the molecular level. We chose from every functional region one representative EA1-causing Kv1.1 mutation: F244C (S1), T284A/M (S2), I320T (S3), L375F (S4), E395D (S4–S5 linker), L399I (S5 N-terminus), S412I/C (S5 C-terminus) and F484C (S6). We tested the electrophysiological properties and disregarded the expression levels in this study since, in vivo, alterations in the expression levels might be compensated. We determined gating and ionic currents in response to a series of depolarizing pulses from −90 mV to voltages between −120 and +80 mV to determine shifts in voltage sensor kinetics, in pore opening and inactivation. We found that the conductance voltage (GV) relationships of all mutants were shifted to less polarized potentials (Figure 1c; Table 2). However, the degree by which the voltage dependence was shifted compared to wildtype (V_½_ = −25 mV) varied significantly from a shift of ΔV_½_ = +5.7 mV (F244C) to ΔV_½_ = +52.9 mV (S412I).

### 2.2. Separation of Voltage Sensor Movement and Pore Opening

A shift of the conductance voltage-relation GV can have several causes. Canonically, the four voltage sensors need to activate before the central ion conducting pore can open. Shifting of the voltage sensor activation (QV) to less polarized potentials would result in a parallel shift of the conductance in the same direction. Alternatively, only the final transitions following voltage sensor activation (e.g., the EA1 mutation V369I in the ILT motif) [40] or open state stabilization [41] might be affected. In this case, only the conductance voltage relation but not the voltage sensor movement would be shifted. Finally, if the energetic coupling strength between voltage sensor movement and pore opening is weakened, i.e., when less energy is transferred from the four peripheral voltage sensing domains to the central pore region, then pore opening (GV) and voltage sensor movement (QV) are separated and shifted to opposite directions [37,38]. Several of the EA1 mutants were located in the area of electromechanical coupling (S4–S5 linker and lower S6; Table 1), and we identified in a previous work I384 and F484 as two of the key residues for electromechanical coupling [38], while E395 is essential for open state stabilization. Therefore, we tested the effect of the mutations on voltage sensor movement. We found that despite the location in the region of electromechanical coupling (Figure 1a), all but L375F (S4) and F484C (S6-C-terminal) showed voltage sensor movement shifted to more positive potentials between +5 and +25 mV. L375F and F484C shifted the voltage sensor movement to more polarized potentials with respect to wildtype by ΔV_½_ = −6 mV and ΔV_½_ = −2 mV, respectively (Figure 1d, Table 2). This shifting of the gating charge movement and pore opening in opposite directions is characteristic for electromechanical uncoupling and is consistent with F484’s role in coupling.

While the QVs of the other EA1 mutants were shifted to more positive potentials, there was no linear relationship between shift of the voltage sensor movement and pore opening (red line in Figure 1d). QV and GV of all mutants but of F244C, whose GV was less shifted, separated by amounts between +5 and +50 mV (indicated by blue lines in Figure 1d). This suggests that in addition to the shift of the voltage sensor movement to less polarized potentials, the later gating transitions preceding pore opening were also significantly affected.

### 2.3. Is Open State Stablization Affected?

Two EA1 mutants (R394T, E395D) alter residues that have previously been identified to form an intersubunit interaction to stabilize the open state of Kv1 [41]. The open state stabilization leads to slow deactivation and manifests itself in a slowed return of the voltage sensors to the resting position [41]. The interaction forms with the S6 of the neighboring subunit (Y485) just neighboring F484, the residue responsible for coupling. The EA1 mutant R394T had been characterized in a previous study by Tristán-Clavijo et al. [12], who found that the GV was shifted to more positive potentials, as all other mutants characterized here, but also that the deactivation kinetics, i.e., the closing speed, was increased. This would be expected according to its role in open state stabilization. We showed previously [41] that the gating currents of E395D lacked open state stabilization and that L398V also abolished the intersubunit interaction. L399I is located in the immediate environment of this interaction. We therefore tested whether open state stabilization was influenced by the EA1 mutants. 

**Figure 2:**
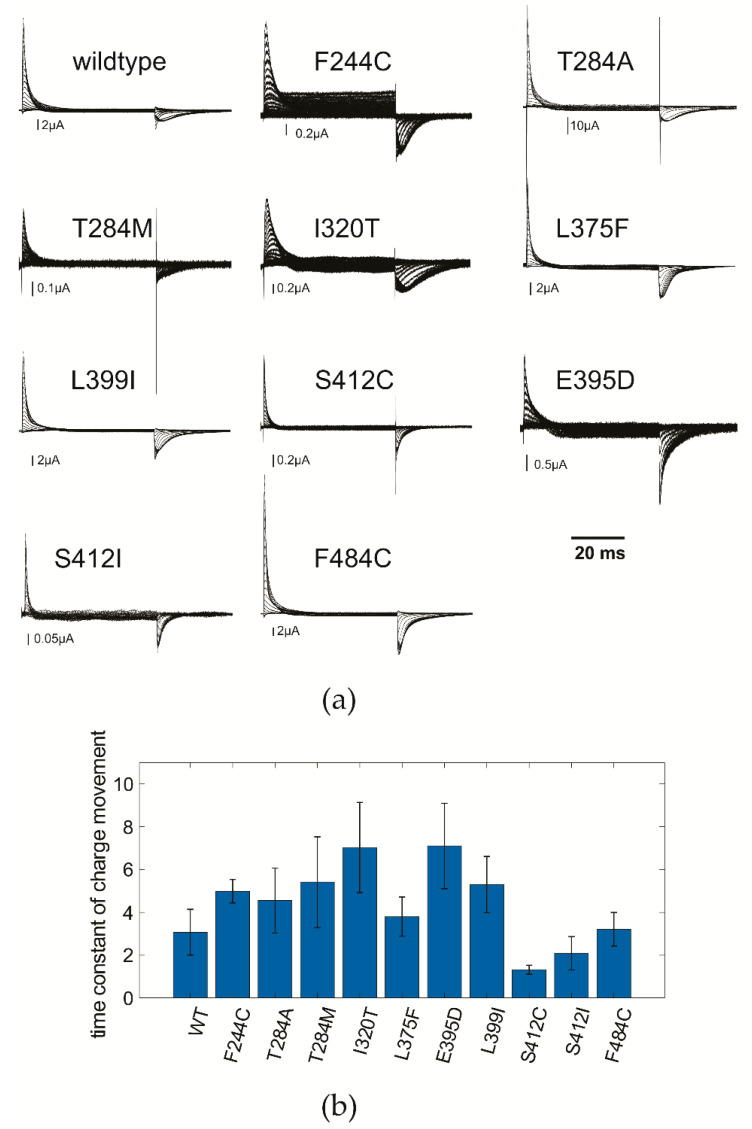
(**a**) Gating currents elicited from wildtype (WT) and EA1 mutants in response to depolarizing pulses from −90 mV to potentials between −120 and +100 mV. F244C and I320T show leak currents in the C-type inactivated W434F mutant indicating impaired C-type inactivation [7]. (**b**) Time constant of gating charge activation at a potential +50 mV more positive than the midpoint of the QV (WT: 0 mV, F244C: 30 mV, T284A: 30 mV, T284M: 20 mV, I320T: 30 mV, L375F: 0 mV, E395D: 10 mV, L399I: 30 mV, S412C: 30 mV, S412I: 20 mV, F484C: 30 mV; *n* = 3–9).

Figure 2a shows the gating currents obtained in the Shaker-W434F mutant. The mutation W434F renders the channel immediately C-type inactivated [42], which allows the measurement of the transient gating currents produced by the movement of the gating charges in the membrane during activation. The wildtype (W434F) channel shows a slowing of the gating charge movement upon deactivation of the channel at depolarizations more positive than −40 mV. This slowing is caused by the stabilization of the open state [41]. Comparison with the gating currents elicited from the EA1 mutants shows that the slowing was absent in the E395D, S412I/C and F484C mutants. The mutant that we suspected L399I as well as the other EA1 mutants showed slowed gating currents during deactivation like the wildtype channel. 

We also investigated whether the activation kinetics, i.e., the speed with which the voltage sensors activate, had been altered. We did not find a consensus in the alteration of the kinetics among the mutants. E395D and I320T were significantly slowed whereas S412I/C were significantly accelerated with respect to wildtype (W434F; Figure 2b).

### 2.4. Noncanonical Coupling in EA1 Mutants

**Figure 3:**
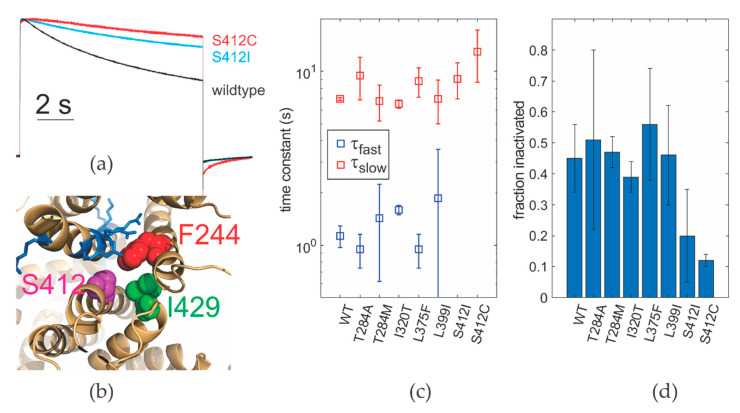
(**a**) Inactivation time courses of wildtype, S412I and S412C determined at 0, +30 and +20 mV, respectively. Current traces were normalized to maximal current. (**b**) Non-canonical coupling. S412C is positioned neighboring F244C and I429. Blue denotes the gating charges in the S4. (**c**) Time constants of inactivation for different EA1 mutants (WT: 0 mV, T284A: 30 mV, T284M: 0 mV, I320T: 60 mV, L375F: 60 mV, L399I: 30 mV, S412I: 60 mV, S412C: 50 mV; *n* = 3–7). (**d**) Fraction of channels inactivated after 8 s (membrane potentials as in (**c**); *n* = 3–7).

We showed previously that the mutation F244C (F184C in hKv1.1) not only shifts activation to less polarized potentials but also couples voltage sensor movement to the pore domain directly via non-canonical coupling [7]. Non-canonical coupling links the extracellular surfaces of voltage sensor and pore [7,14,43,44] and occurs in addition to the canonical pathway that links both domains via the S4–S5 linker, annealed to the distal S6. The non-canonical coupling has also been suggested to be significant for activation of the non-domain swapped Kv channels where the S4–S5 linker is very short [44]. In Petitjean et al. [7], we showed that the F244C mutation in the S1 interacts directly with the central selectivity filter of the Kv channel. We proposed that the interaction was transferred from F244 via I429 to the selectivity filter, where it inhibits C-type inactivation. The EA1 mutations S412I/C (S342I/C in hKv1.1) are located in the S5 helix in immediate proximity to F244 and I429 (Figure 3b), thus likely influencing the same interaction. It is therefore possible that the non-canonical coupling path and influence of C-type inactivation play a role in other mutations linked to EA1 development. We determined the speed of inactivation and the residual current after 8 s inactivation (Figure 3). Again, we corrected for the shift in the conductance voltage relation of the mutants with respect to wildtype by determining inactivation at +30 mV above the midpoint of the GV. While most mutants did not alter C-type inactivation at the respective membrane potential with respect to wildtype, the two mutants at the non-canonical coupling path (S412I/C) inactivated significantly slower than the wildtype (Figure 3a). The faster of the two inactivation time constants was absent in these channels (Figure 3c). In addition, the fraction of channels that did inactivate in an 8 s interval was significantly lower (Figure 3d, Table 3). Only 10–20% of the channels inactivated in the time interval of 8 s compared to 45–65% for the wildtype. A lower percentage of inactivated channels was also observed for other mutants that were tested, but here, the effect was less significant (Figure 3d). It is also known that the expression level influences the percentage of inactivated channels, so we have to be careful when comparing the different mutants. 

While C-type inactivation was not directly altered in most EA1 mutants, we should still consider its consequence. Here, we compared time constants normalized to the midpoint of activation. However, the robust shift of the voltage dependence of opening to less polarized potentials will lead to slower C-type inactivation when considering a specific membrane potential.

## 3. Discussion

In this work, we studied the electrophysiological properties of representative EA1 mutants in Kv1.1. The rationale was that the concentrations of the mutations in either the voltage sensor or the region for electromechanical coupling suggested that a common principle is causal for the development of episodic ataxia. It was striking that basically all major gating mechanisms that have been intensely studied over the last decades in the Kv channels can be linked to one or more EA1 mutations as we showed above. This includes electromechanical coupling (L375F, F484C), open pore stabilization by the intersubunit interaction RELY (E395D, R394T), the non-canonical gating between voltage sensor and pore (F244C, S412I/C), C-type inactivation and pore opening. While each of these mechanisms by themselves are fascinating from the biophysical point of view, the diverse alteration of these mechanisms among the mutants meant that they were not the common cause for generation of episodic ataxia.

The one parameter that was altered in all mutants that we studied was a shift of the conductance voltage relation to less polarized potentials. With this shift in conductance, also the activation, deactivation and inactivation were shifted to depolarized potentials. Such a shift of the conductance voltage relation to less polarized potentials had been previously reported for other EA1 mutations including R297T, F373V, R377F, G381D, V478A and F484S [8,9,10,11,12]. We should mention that the experiments were carried out at room temperature, as it is customary for Xenopus oocytes. Increasing the temperature by ~10–15 °C to 37 °C will slightly accelerate all processes. This would be true for wildtype as well as all EA1 mutants. While the speed of the Shaker channel depends on temperature, its voltage-dependence of activation, i.e., the relative energies of closed and open state, are only slightly affected by temperature [45,46]. Not all single point mutations will affect the temperature dependence equally. It has been shown that the polarity of those residues that alter their solvent exposure during channel activation alters the temperature dependence of channel activation [47]. Although the degree of shift due to increased temperature might vary among the EA1 mutants with respect to wildtype, it is likely that the overall effect of shifting the conductance voltage relation is not affected.

Several mutants have been reported to express to a lower level, and we found the S412I/C mutants show low expression rates in this study. A lower expression rate and a conductance voltage relation shifted to less polarized potentials will have similar effects. Both will result in lower K^+^ currents for a given membrane potential. A lower expression rate might, however, be compensated by regulatory mechanisms by the second copy of the gene or by other remodeling mechanisms. 

**Figure 4:**
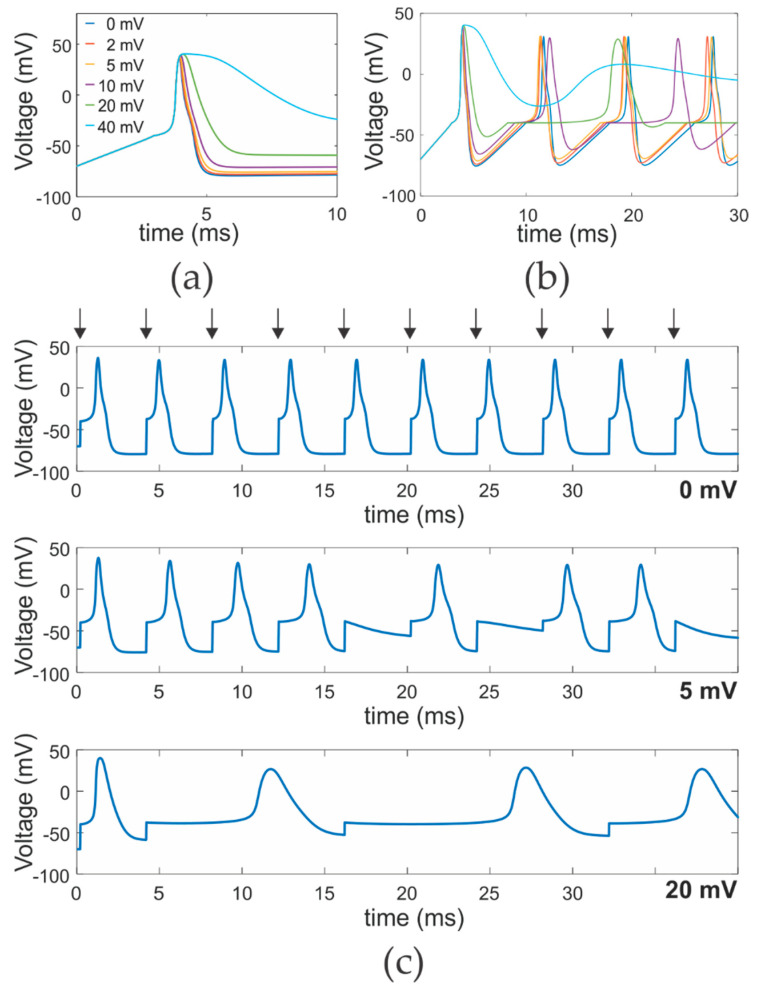
(**a**) Simulation of a single action potential according to a Hodgkin and Huxley model, where the voltage dependence of the voltage-gated potassium channels has been shifted by the amount indicated in the legend. (**b**) Simulation of a train of action potentials in response to continuous current stimulation (colors as in (**a**)). (**c**) Simulation of a train of action potentials in response to a train of current stimulations (indicated by arrows on top). Kv activation has been shifted by 0 mV (top), 5 mV (center) and 20 mV (bottom).

The main role of Kv1.1 is the repolarization of action potentials after excitation in the axon. The shift in conductance voltage relation to depolarized potentials leads to activation delayed to the onset of the action potential. In addition, the mutated Kv1.1 will close more rapidly thereby further prolonging the action potentials. To better understand the effect that a shift in the activation of Kv channels has on the form of the action potentials, we simulated the form of an action potential based on the Hodgkin and Huxley model [48] (Figure 4a). Already a shift of 2 mV prolongs the action potential, and the duration increases continuously until, at extreme cases (40 mV, T284M, I320T, L375F, L399I), the repolarization becomes extremely slow, mainly carried by the leak currents. In vivo, this would probably be compensated by upregulation of other Kv channels such as Kv1.2 and Kv1.4. 

For a single action potential, the exact duration is not decisive, as it will still be propagated along the axon. The duration plays a role for trains of action potentials. The “remaining” membrane depolarization as well as the K^+^ conductance will set the excitability of the membrane. We tested how trains of action potentials would be affected by continuous stimulation (current injection, Figure 4b) or repeated short stimuli with a frequency of 250 Hz (Figure 4c). The frequency of the action potentials in response to a continuous stimulus increases slightly with small shifts in the Kv conductance voltage relation (<10 mV). Here, the lower K^+^ conductance of the membrane increases excitability. At larger shifts, and consequently longer action potential duration, the frequency decreased. This was caused by deeper inactivation of the sodium channels. When the Kv channels activate delayed and close prematurely, the repolarization is to a large part carried by inactivation of the sodium channels. Accordingly, at the beginning of the next action potential less sodium channels remain available and eventually compete against the counteracting potassium channels. This also led to missed firing in response to trains of action potentials (Figure 4c).

Our simulations were based on the original Hodgkin and Huxley model and did not include inactivation of the potassium channels. Inactivation of Kv1 channels is also the parameter where the consequences of a shifted conductance voltage relation diverges from the effect of a lower expression. Although, for a given voltage, the ionic current might be identical, the voltage dependence of entry into inactivation will be shifted with the activation of the channel. For this reason, less channels will inactivate, and this reduced inactivation will play a role at high firing rates similar to delayed channel activation and, consequently, the prolonged action potential duration. 

C-type inactivation was directly affected by the three mutants in the non-canonical coupling path between the S1 and the pore [7]. Here, C-type inactivation was slowed down and less complete, in addition to the shift in the conductance voltage relation. Under normal conditions, the repeated activation of the membrane will lead to adaptation of the excitability by accumulation of inactivated Kv channels. This adaptation of the excitability will be reduced when less channels inactivate. In those EA1 mutants that alter inactivation, the varied accumulative effect of C-type inactivation would be even more strongly expressed.

Setting aside how accurately models of action potentials can predict the effect in a neuron, the question remains how the observed effects on the biophysical properties lead to the development of episodic ataxia. As mentioned above, both the delayed activation and the resulting prolonged action potentials and the lack of adaptation to high firing rates due to impaired C-type inactivation play a role whenever the period between action potentials is diminished. This might explain why episodic ataxia occurs only episodically and is triggered by physical or mental stress or startle. To better understand how the molecular mechanisms are linked to development of episodic ataxia, the effects of the ataxia mutations need to be investigated in situ. Bergum et al. [49], for instance, have studied the effect of the Kv1.1-V408A (V478A in Shaker) mutant in a transgenic mouse and found that the action potentials in the basket cell terminals are prolonged, just as would be predicted by a shift in the conductance voltage relation. They also found lower frequency in spontaneous Purkinje cell firing, just as would be predicted for lower excitability. Both results agree with our simulations. Now that we understand the molecular mechanisms of the different EA1 mutants, we can use this toolset of mutants that variably alter specific gating parameters to study their effect directly in neurons or acute brain slices. We will be able to study the effect of the different gating mechanisms on excitability, action potential shapes, synaptic plasticity and signal transduction. Introducing L375F would, for instance, move gating charge movements in the opposite direction than I320T, with a similar shift in the GV. Similarly, we will be able to distinguish between a lower expression and a shifted GV. We envision that these future studies will help us to track down the mechanism by which the ataxia is triggered during an episode and, in the future, design a pharmacological treatment strategy. 

## 4. Materials and Methods 

### 4.1. Molecular Biology

Mutations were introduced into a plasmid encoding the Drosophila Shaker gene H4 in the pBSTA vector with N-type inactivation removed by deletion of amino acids 6–46 [50]. Channels to measure gating currents also carried the W434F mutation. Point mutations were generated using site-directed mutagenesis (QuikChange; Stratagene, San Diego, CA, USA), and correct insertion of the mutation was verified by sequencing. Oocytes were surgically removed from Xenopus laevis following the guidelines of the university’s Ethics Committee for Animal Experimentation (CDEA). Follicular membrane was enzymatically removed by collagenase treatment. RNA was in vitro transcribed (mMachine T7, Invitrogen, Carlsbad, CA, USA), and 46 nl RNA was injected into each oocyte at a concentration of 0.1–1 µg/µL using a nano-injector (Drummond Scientific, Broomall, PA, USA). Injected oocytes were incubated in Barth medium supplemented with 5% horse serum at 18 °C for 1–3 days before recordings.

### 4.2. Electrophysiological Recordings

Electrophysiological recordings were performed with the cut-open oocyte voltage clamp technique for spatial voltage homogeneity and fast temporal resolution [41,51]. Currents were recorded using “GPatch” acquisition software. Oocytes were placed in a 3-part chamber; top, middle, bottom, containing an external solution (115 mM N-methyl-D-glucamine (NMDG), 10 mM HEPES, 2 mM Ca(OH)_2_) adjusted to pH 7.1 using methane-sulfonic acid (MES). The membrane exposed to the bottom chamber was permeabilized by exchanging external solution in the bottom chamber with 0.2% saponin so the current could be injected directly into the oocyte. Saponin is replaced by an internal solution (10 mM HEPES, 2 mM EDTA, 115 mM NMDG (for gating current recording) or KOH (for ionic current recording)) adjusted to pH 7.1 using MES. The voltage electrode was filled with 3 M KCl. Recordings were done at room temperature.

### 4.3. Analysis

Conductance (*G*) was calculated from the steady state currents (1) via

(1) $$G=\frac{I}{V-V_{rev}}$$

where *V_rev_* is the reversal potential or from tail currents when pulsing to intermediate potentials at the end of the depolarizing pulse. Conductance-voltage relationships (*GV*) were fitted to a Boltzmann relation of the form (2)

(2) GGmax=(1+exp(−zappF(V−V12)RT))−1

where *G_max_* denotes the maximal conductance, *z_app_* the apparent gating charge, *F* the Faraday constant, *R* the universal gas constant and *T* the temperature in K.

The total gating charge (*Q*) was calculated by integrating the transient gating currents, and charge-voltage relationships (*QV*) where fitted to (3)

(3) QQmax=(1+exp(−zappF(V−V12)RT))−1

where *Q_max_* denotes the maximal gating charge.

Activation, deactivation and inactivation time constants were obtained by fitting the time course of ionic current to a sum of 2 or 3 exponentials. To compensate for shifts of the GV to less polarized potentials, the time constants for each mutant were obtained at a voltage calibrated to the V_½_ of the GV.

Images of 3D structures were produced using Pymol.

### 4.4. Simulation of Neuronal Action Potentials

Neuronal action potentials were simulated based on a Hodgkin and Huxley [48] model. Each voltage sensor was described by a two state Markov model. Activation of Kv and Nav channels require four and three identical voltage sensors to open, respectively. A fourth voltage sensor governs Nav inactivation. Nav and Kv channel opening was determined by numerical integration of the Markov models in 2 µs time steps. Changes in membrane potential were calculated from the conductance and membrane capacitance. All simulations were done in Matlab (Mathworks). 

## Figures and Tables

**Table 1:** List of mutations in Kv1.1 linked to episodic ataxia type 1. Mutants with star * have been studied in this manuscript.

Position in hKv1.1	This Study	Position in Shaker Kv	Domain	Functional Region	Reference
R167M		R227M	S1	VSD ^1^	[16]
A170S		A230S	S1	VSD	[17]
V174F		V234F	S1	VSD	[18]
I176R		I236R	S1	VSD	[19]
I177N		I237N	S1	VSD	[20]
F184C	*	F244C	S1	VSD	[21]
C185W		C245W	S1	VSD	[16]
T226R		T284R	S2	VSD	[22]
T226A	*	T284A			[19]
T226M	*	T284M			[23]
T226K		T284K			
R239S		R297S	S2	VSD	[18]
F249I		F307I	S3	VSD	[18]
F249C		F307C			
I262T	*	I320T	S3	VSD	[24]
I262M		I320M			[25]
E283K		–	S3–S4 linker	VSD	[26]
V299I		V369I	S4	VSD	[27]
				(ILT motif)	
F303V		F373V	S4	VSD	[10]
L305F	*	L375F	S4	VSD	[28]
R307S		R377F	S4	VSD	[8]
G311S		G381S	S4–S5 linker	EMC ^2^	[21]
G311D		G381D			
I314T		I384T	S4–S5 linker	EMC	[29]
R324T		R394T	S4–S5 linker	EMC	[12]
E325D	*	E395D	S4–S5 linker	EMC	[21]
L329I	*	L399I	S5	EMC	[30]
S342I	*	S412I	S5	Pore	[31]
S342C	*	S412C			
P403S		P473S	S6	PVP motif	[32]
V404I		P474I	S6	PVP motif	[19]
I407M		I477M	S6	EMC	[16]
V408A		V478A	S5	EMC	[18]
V408L		V478L			[33]
F414C	*	F484C	S6	EMC	[34]
F414S		F484C	S6	EMC	[8]

^1^ Voltage Sensing Domain, ^2^ Electromechanical Coupling Region.

**Table 2:** Voltage dependence of gating charge movement and conductance of the EA1 mutants.

Position	Charge Movement ^1^		Conductance	
	V_½_ (mV)	z_app_ (*e*)	V_½_ (mV)	z_app_ (*e*)
Wildtype ^1^	−45.3 ± 1.4	2.6 ± 0.3	−25.2 ± 1.4	3.7 ± 0.5
F244C	−24.8 ± 2.5	3.0 ± 0.5	−19.5 ± 0.7	2.6 ± 0.1
T284A	−22.9 ± 1.9	1.7 ± 0.1	2.2 ± 2.4	1.7 ± 0.2
T284M	−19.8 ± 1.3	1.7 ± 0.1	14.6 ± 0.8	1.8 ± 0.1
I320T	−18.9 ± 2.9	1.5 ± 0.1	18.7 ± 0.4	1.4 ± 0.1
L375F	−51.5 ± 0.3	2.4 ± 0.2	18.5 ± 1.8	1.4 ± 0.1
E395D	−40.8 ± 0.7	2.2 ± 0.1	−14.9 ± 1.8	2.4 ± 0.4
			11.0 ± 1.8	0.5 ± 0.1
L399I	−42.3 ± 1.0	2.1 ± 0.1	19.3 ± 1.0	1.3 ± 0.1
S412I S412C	−33.3 ± 0.7 −33.7 ± 0.7	2.6 ± 0.2 2.6 ± 0.2	27.7 ± 1.5 5.6 ± 0.4	1.1 ± 0.1 1.8 ± 0.5
F484C	−47.1 ± 0.5	3.2 ± 0.2	−6.0 ± 2.8 ^2^	1.3 ± 0.2
			50.0 ± 6.6	2.6 ± 0.5

^1^ The mutant W434F was used for all mutants including wildtype to determine charge movement. ^2^ F484C and E395D conductance voltage relations were fit with a sum of two Boltzmann distributions.

**Table 3:** Inactivation time constants of the EA1 mutants.

Position	Inactivation		
	τ_fast (ms)_	τ_slow (ms)_	% Inact.
Wildtype 1.1 ± 0.2	6.9 ± 0.1	45 ± 11	
T284A	1.0 ± 0.2	9.5 ± 2.6	51 ± 29
T284M	1.4 ± 0.8	6.8 ± 1.6	47 ± 5
I320T	1.6 ± 0.1	6.5 ± 0.4	39 ± 5
L375F	1.0 ± 0.2	8.8 ± 1.7	56 ± 18
S412I	–	9.1 ± 2.1	20 ± 15
S412C	–	13.0 ± 4.3	12 ± 2

Values marked—did not exist.
